# Genome-wide identification and comparative analysis of DNA methyltransferase and demethylase gene families in two ploidy *Cyclocarya paliurus* and their potential function in heterodichogamy

**DOI:** 10.1186/s12864-023-09383-5

**Published:** 2023-05-29

**Authors:** Qian Wang, Yinquan Qu, Yanhao Yu, Xia Mao, Xiangxiang Fu

**Affiliations:** 1 Co-Innovation Center for Sustainable Forestry in Southern China, College of Forestry, Nanjing Forestry University, Nanjing, 210037 China; 2 Fishery College, Zhejiang Ocean University, Zhoushan, 316022 Zhejiang China; 3 Jiangsu Vocational College of Agriculture and Forestry, Zhenjiang, 212400 Jiangsu China

**Keywords:** DNA methylase, DNA demethylase, Polyploidization, Heterodichogamy

## Abstract

**Background:**

DNA methylation is one of the most abundant epigenetic modifications, which plays important roles in flower development, sex differentiation, and regulation of flowering time. Its pattern is affected by cytosine-5 DNA methyltransferase (C5-MTase) and DNA demethylase (dMTase). At present, there are no reports on *C5-MTase* and *dMTase* genes in heterodichogamous *Cyclocarya paliurus*.

**Results:**

In this study, 6 *CpC5-MTase* and 3 *CpdMTase* genes were identified in diploid (2n = 2 ×  = 32) *C. paliurus*, while 20 *CpC5-MTase* and 13 *CpdMTase* genes were identified in autotetraploid (2n = 4 ×  = 64). 80% of identified genes maintained relatively fixed positions on chromosomes during polyploidization. In addition, we found that some DRM subfamily members didn’t contain the UBA domain. The transcript abundance of *CpC5-MTase* and *CpdMTase* in male and female flowers of two morphs (protandry and protogyny) from diploidy was analyzed. Results showed that all genes were significantly up-regulated at the stage of floral bud break (S2), but significantly down-regulated at the stage of flower maturation (S4). At S2, some *CpC5-MTase* genes showed higher expression levels in PG-M than in PG-F, whereas some *CpdMTase* genes showed higher expression levels in PA-M than in PA-F. In addition, these genes were significantly associated with gibberellin synthesis-related genes (e.g. *DELLA* and *GID1*), suggesting that DNA methylation may play a role in the asynchronous floral development process through gibberellin signal.

**Conclusions:**

These results broaden our understanding of the *CpC5-MTase* and *CpdMTase* genes in diploid and autotetraploid *C. paliurus*, and provide a novel insight into regulatory mechanisms of DNA methylation in heterodichogamy.

**Supplementary Information:**

The online version contains supplementary material available at 10.1186/s12864-023-09383-5.

## Background

As a conserved epigenetic modification, DNA methylation was widely found in plants and animals, which can alter gene expression and phenotype without altering DNA sequence and maintain genomic stability [1–3]. DNA methylation is controlled by a series of cytosine-5 DNA methyltransferase (C5-MTase) and DNA demethylase (dMTase). In plants, DNA methylation commonly occurs at symmetrical CG and CHG sequence context, and asymmetrical CHH context (H = A, T, or C), which can be maintained or established de novo DNA methylation by distinct pathway [1]. Generally, methylation at the CG and CHG contexts is maintained by methyltransferase 1 (MET1) and chromomenthylase 2/3 (CMT2/3), while methylation at the CHH context is maintained by CMT2 and domain rearranged methyltransferase 2 (DRM2) [4, 5]. Among these, DRM catalyzes de novo methylation via the RNA-directed DNA methylation (RdDM) pathway in both symmetric and asymmetric sequence contexts [2, 4, 6]. CMT is a plant-specific C5-MTase, containing bromo-adjacent homology (BAH) and chromo domain bound to histone H3K9 to maintain DNA methylation [2]. DNA methyltransferase 2 (DNMT2) is the smallest methyltransferase in eukaryotes, which can methylate tRNA(Asp)C38 in vitro [7].

DNA methylation level is dynamically regulated depending not only on the establishment and maintenance of DNA methylation but also on DNA demethylation. DNA methylation can be passively removed by inhibiting C5-MTase activity in DNA replication or actively removed by a series of demethylases [2, 4, 8]. The dMTase proteins can be classified into four subfamilies in *Arabidopsis thaliana*, including transcriptional activator demeter (DME), repressor of silence 1 (ROS1), demeter-like 2 (DML2), and demeter-like 3 (DML3) [9]. As the necessity for the establishment of genomic imprinting in the endosperm, DME mainly expresses in central cells; whereas ROS1 and DML2/3 express widely in all vegetative tissues [10–12]. Furthermore, ROS1 can inhibit the methylation of gene promoters and transposons [13], and DML2/3 can protect genes from potentially deleterious methylation [14].

*Cyclocarya paliurus*, also known as “*sweet tea*”, is a medicinal plant belonging to the family Juglandaceae [15]. *C. paliurus* is rich in triterpenes, flavonoids, polysaccharides, polyphenols, and other active substances, which have hypoglycemic and hypotensive functions. The attractive ingredients in its leaves have led to massive plantations for leaf-use in China. However, heterodichogamy in *C. paliurus* seems to slow down its popularization due to low seed fullness, which has been found in half species of Juglandaceae [16]. As a transitional type from dichogamy to dioecy, heterodichogamy possesses two mating types: protogyny (PG) and protandry (PA). Opposite-sex flowers of the intra-mating type are asynchronized in flowering, whereas these of the inter-mating type are synchronized [17]. Supposedly, DNA methylation could regulate flowering by affecting the expression of flowering-related genes. For example, the changes in methylation status of *MeGI* caused the reversible sex determination in *Diospyros kaki* [18]. In *Arabidopsis thaliana*, the reduction of genomic methylation level promoted flowering by suppressing *FLC* expression; while in cotton, the inhibition of DNA methylation promoted flowering by activating the *COL2* expression [19, 20]. Campos-Rivero et al. [21] showed that epigenetic regulates hormone signaling to inhibit or promote flowering in angiosperms, such as gibberellin, auxin, and ethylene. Moreover, plant hormones can alter DNA methylation status in specific and redundant ways [22]. Previous research has indicated that gibberellin, especially GA_3_, plays an important role in asynchronous floral development in heterodichogamous *C. paliurus* [23]. However, the regulatory mechanism of DNA methylation in asynchronous floral differentiation and development in heterodichogamous *C. paliurus* is still unclear. Additionally, new findings indicated that minority diploid and majority autotetraploidy co-exist in nature [17]. Thus, heterodichogamy coupled with an autotetraploid complicates the issue of seed setting in *C. paliurus*. Fortunately, whole genomes of two ploidy *C. paliurus* (PG and PA in diploidy and PA in autotetraploidy) have been published, revealing that the evolving process of autotetraploid experienced three whole-genome duplications (WGD) [17]. It is also well known that DNA methylation plays an important role in the adaptation of polyploid plants to WGD [24, 25]. For instance, autotetraploid rice has been shown to reduce deleterious genomic dosage effects by increasing the methylation of class II transposable elements [25].

C5-MTases and dMTases gene families with species specificity have been identified in numerous plants [4, 9, 12, 26]. Moreover, gene duplication and loss events constitute the main factors driving the evolution of these two gene families [27]. In *C. paliurus*, we hypothesized that: 1) C5-MTase and dMTase gene families undergo gene expansion or loss events during polyploidization; and 2) heterodichogamous character is regulated by methylation of related genes. To verify these hypotheses, our work focused on: 1) identifying and analyzing all members of C5-MTase and dMTase gene families by screening whole genomes of autotetraploid and its diploid ancestors; and 2) comparatively analyzing the expression profiles of two gene families in both male and female flowers between two mating types (PA and PG) from diploid plants. This study may lay the foundation for further research on the role of DNA methylation in *C. paliurus*.

## Results

### Genome-wide identification and structural analysis of *C5-MTase* and *dMTase* genes in* C. paliurus*

In our study, we identified 26 *C5-MTase* and 16 *dMTase* genes from 2 genomes of *C. paliurus* based on HMM and Blastp analyses. Among them, 6 *CpC5-MTase* and 3 *CpdMTase* genes were identified in diploid *C. paliurus*, while 20 *CpC5-MTase* and 13 *CpdMTase* genes were identified in autotetraploid *C. paliurus*. The *CpC5-MTase* genes in diploid included 1 *MET* (*CpMET-D1*), 2 *CMT* (*CpCMT2-D1* and *CpCMT3-D1*), 1 *DNMT2* (*CpDNMT2-D1*), and 2 *DRM* (*CpDRM-D1/2*), while 7 *CMT* (*CpCMT2-T1/2/3/4* and *CpCMT3-T1/2/3*), 4 *MET* (*CpMET-T1/2/3/4*), 6 *DRM* (*CpDRM-T1/2/3/4/5/6*), and 3 *DNMT2* (*CpDNMT2-T1/2/3*) were identified in autotetraploid. The results found that the protein length of 26 CpC5-MTase varied from 393 (*CpDNMT2-D1*) to 1578 (*CpMET-T2*) amino acids (aa), and their predicted molecular weights ranged from 44,309.27 to 177,676.64 kDa. The PI values spanned from 5.26 to 9.01; the subfamily DNMT2 was deemed the basic proteins (PI > 7) (Table 1). The grand average of hydropathicity (GRAVY) values ranged from -0.606 to -0.338, indicating that all CpC5-MTase proteins were hydrophilic. Most of the proteins were predicted to be located in the cell nucleus, except for *CpDRM-D2*, *CpCMT2-T2*, and *CpDRM-T5*, which were predicted to be in the cytoplasm.

**Table 1:** Basic information of *C5-MTase* and *dMTase* genes in diploid and autotetraploid *C. paliurus*

Gene Name	Gene ID	ORF (bp)	Amino acid (aa)	Molecular weight (kDa)	PI	GRAVY value	No. of intron	Subcellular predicted
Cytosine-5 DNA methyltransferases in diploid								
* CpCMT2-D1*	CpaM1st29183	3102	1033	117,470.2	8.01	-0.506	21	Nucleus
* CpCMT3-D1*	CpaM1st09643	2511	836	94,031.35	5.36	-0.491	20	Nucleus
* CpMET-D1*	CpaM1st19889	4620	1539	173,555.03	5.99	-0.491	11	Nucleus
* CpDRM-D1*	CpaM1st24345	1866	621	69,282.00	5.26	-0.437	10	Nucleus
* CpDRM-D2*	CpaM1st34737	1335	444	50,378.73	7.59	-0.344	3	Cytoplasm
* CpDNMT2-D1*	CpaM1st31201	1182	393	44,309.27	7.2	-0.367	9	Nucleus
Demethylases in diploid								
* CpROS1-D1*	CpaM1st40636	5370	1789	200,763.8	5.9	-0.709	19	Nucleus
* CpDME-D1*	CpaM1st11558	5811	1936	217,279.43	6.24	-0.745	18	Nucleus
* CpDME-D2*	CpaM1st16411	6021	2006	224,642.71	6.94	-0.709	19	Nucleus
Cytosine-5 DNA methyltransferases in autotetraploid								
* CpCMT2-T1*	C.paliurus-tetra126170	2151	716	81,100.06	6.22	-0.386	17	Nucleus
* CpCMT2-T2*	C.paliurus-tetra126184	1557	518	58,636.49	5.45	-0.338	11	Cytoplasm
* CpCMT2-T3*	C.paliurus-tetra126196	3744	1247	141,441.60	9.01	-0.589	23	Nucleus
* CpCMT2-T4*	C.paliurus-tetra129077	3666	1221	138,414.73	8.92	-0.606	22	Nucleus
* CpCMT3-T1*	C.paliurus-tetra023585	2511	836	93,966.29	5.29	-0.478	20	Nucleus
* CpCMT3-T2*	C.paliurus-tetra028905	2502	833	93,671.99	5.33	-0.48	20	Nucleus
* CpCMT3-T3*	C.paliurus-tetra031373	2511	836	93,964.32	5.33	-0.482	20	Nucleus
* CpMET-T1*	C.paliurus-tetra065029	4719	1572	176,877.80	5.99	-0.485	11	Nucleus
* CpMET-T2*	C.paliurus-tetra066914	4737	1578	177,676.64	6.03	-0.503	11	Nucleus
* CpMET-T3*	C.paliurus-tetra066921	4635	1544	173,700.16	5.86	-0.484	11	Nucleus
* CpMET-T4*	C.paliurus-tetra069029	4731	1576	177,380.33	5.99	-0.497	11	Nucleus
* CpDRM-T1*	C.paliurus-tetra095687	4596	1531	168,826.34	6.62	-0.536	20	Nucleus
* CpDRM-T2*	C.paliurus-tetra098975	1854	617	69,001.73	5.26	-0.43	10	Nucleus
* CpDRM-T3*	C.paliurus-tetra103779	1938	645	72,118.20	5.33	-0.429	10	Nucleus
* CpDRM-T4*	C.paliurus-tetra145580	4065	1354	149,523.38	6.58	-0.579	18	Nucleus
* CpDRM-T5*	C.paliurus-tetra152058	1335	444	50,392.80	7.99	-0.345	3	Cytoplasm
* CpDRM-T6*	C.paliurus-tetra155575	1869	622	69,844.98	4.99	-0.377	7	Nucleus
* CpDNMT2-T1*	C.paliurus-tetra131472	2508	835	94,308.53	7.03	-0.435	12	Nucleus
* CpDNMT2-T2*	C.paliurus-tetra131491	2202	733	83,035.64	8.87	-0.443	16	Nucleus
* CpDNMT2-T3*	C.paliurus-tetra142017	2202	733	83,035.64	8.87	-0.443	16	Nucleus
Demethylases in autotetraploid								
* CpROS1-T1*	C.paliurus-tetra162062	5370	1789	200,617.69	5.91	-0.705	17	Nucleus
* CpROS1-T2*	C.paliurus-tetra165368	5364	1787	200,408.45	5.92	-0.708	18	Nucleus
* CpROS1-T3*	C.paliurus-tetra169007	5370	1789	200,700.68	5.85	-0.711	17	Nucleus
* CpROS1-T4*	C.paliurus-tetra171692	5355	1784	200,250.16	5.83	-0.716	19	Nucleus
* CpDME-T1*	C.paliurus-tetra026026	5862	1953	219,468.22	6.76	-0.769	17	Nucleus
* CpDME-T2*	C.paliurus-tetra028439	5865	1954	219,489.27	6.66	-0.775	13	Nucleus
* CpDME-T3*	C.paliurus-tetra028446	5685	1894	212,862.34	6.73	-0.809	16	Nucleus
* CpDME-T4*	C.paliurus-tetra030774	8004	2667	301,133.41	6.63	-0.878	16	Nucleus
* CpDME-T5*	C.paliurus-tetra047265	5979	1992	223,203.02	6.87	-0.717	19	Nucleus
* CpDME-T6*	C.paliurus-tetra047268	5979	1992	223,172.99	6.93	-0.718	19	Nucleus
* CpDME-T7*	C.paliurus-tetra049582	5982	1993	222,991.70	6.99	-0.736	19	Nucleus
* CpDME-T8*	C.paliurus-tetra051986	5892	1963	219,874.05	6.87	-0.738	19	Nucleus
* CpDME-T9*	C.paliurus-tetra054350	5940	1979	221,647.24	6.93	-0.724	18	Nucleus

*ORF* Open reading frame, *pI* Theoretical isoelectric point, *GRAVY* Grand average of hydrophobicity

Sixteen *CpdMTase* contained 2 *DME* (*CpDME-D1/2*) and 1 *ROS1* (*CpROS1-D1*) in diploid, 9 *DME* (*CpDME-T1/2/3/4/5/6/7/8/9*) and 4 *ROS1* (*CpROS1-T1/2/3/4*) in autotetraploid, respectively. The sequence characteristic analysis showed that the protein length of CpdMTase varied from 1784 (*CpROS1-T4*) to 2667 (*CpDME-T4*) aa, the predicted molecular weights were from 200,250.16 to 301,133.41 kDa, and the PI values ranged from 5.83 to 6.94. The GRAVY values of all proteins were < 0, indicating that CpdMTase proteins were all acidic (PI < 7) and hydrophilic. Putative subcellular localization revealed that all CpdMTase proteins localized to the nucleus (Table 1).

**Fig. 1:**
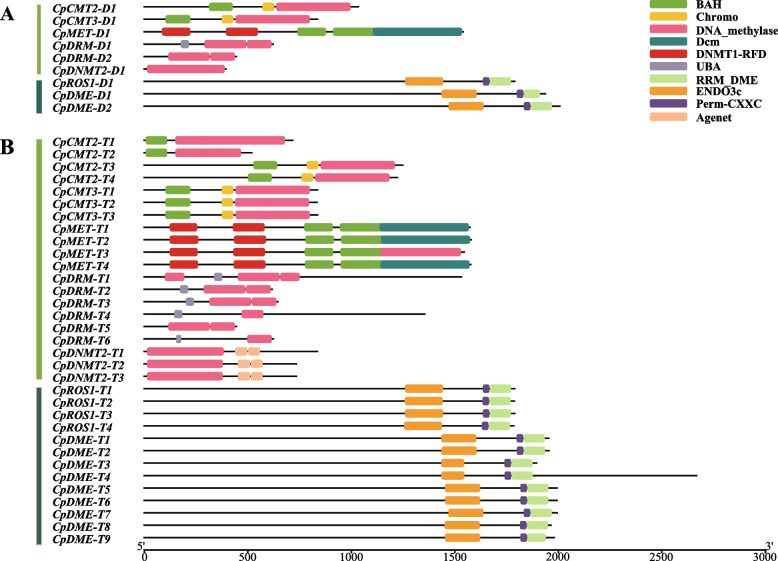
Conserved domain analysis of CpC5-MTase and CpdMTase in diploid (**A**) and autotetraploid (**B**) *C. paliurus*. The different colored boxes indicate different conserved domains and their positions in each protein sequence

Based on the results of structural domain analysis (Fig. 1), the N-terminus of CpC5-MTase proteins in each subfamily showed different combinations of conserved domains. However, no difference in the structure of C5-MTase proteins was found between the two genomics, except that the average length in autotetraploid (999 aa) was greater than that in diploid (811 aa) (Table 1). CpMETs contained 2 DNMT1-RFD domain and 2 BAH domain (Fig. 1), while CpCMTs contained 1 BAH domain, and CpDNMT2s had no N-terminus. Most CpDRMs contained 1 UBA domain, but both CpDRM-D2 and CpDRM-T5 possessed an extra incomplete DNA methylase domain instead of a UBA domain. The RRM-DME, PERM-CXXC, and ENDO3c domains (including the HhH-GPD domain) appeared in all CpdMTases, which were also reported in AtdMTases (Fig. S1). Similarly, the average length of CpdMTases in autotetraploid (1964 aa) was greater than that in diploid (1910 aa) (Table 1).

### Phylogenetic analysis of C5-MTase and dMTase in *C. paliurus* and other plant species

**Fig. 2:**
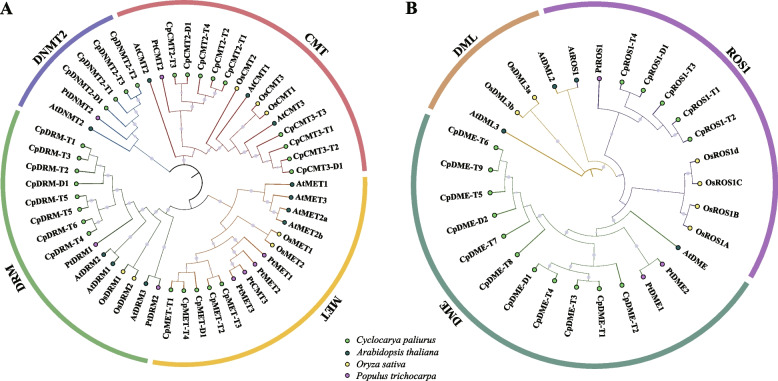
Phylogenetic analysis of the C5-MTase (**A**) and dMTase (**B**) proteins. Cp, *Cyclocarya paliurus*; At, *Arabidopsis thaliana*; Os, *Oryza sativa*; Pt, *Populus trichocarpa*

To further explore the evolutionary relationship between *CpC5-MTase* and *CpdMTase*, a phylogenetic tree was constructed based on the protein sequences of C5-MTase and dMTase from typical monocots (*Oryza sativa*), dicots (*A. thaliana*), and woody species (*Populus trichocarpa*) (Fig. 2). Obviously, the phylogenetic tree showed that C5-MTase families from the 4 species were naturally grouped into 4 categories, namely CMT, MET, DRM, and DNMT2, respectively (Fig. 2A). The DNMT2 category, with 6 genes, was the smallest in the phylogenetic tree, including AtDNMT2, PtDNMT2, CpDNMT-D1, and CpDNMT-T1/2/3. Twenty-nine dMTase proteins were divided into 3 categories, namely DME, ROS1, and DML, respectively (Fig. 2B), in which ROS1s and DMLs showed a closer relationship. In addition, CpC5-MTase and CpdMTase proteins sequences showed high conservation in diploid *C. paliurus*, autotetraploid *C. paliurus*, *A. thaliana*, and *P. trichocarpa*, indicating their similar functions in different species.

### Chromosomal location and synteny analysis

**Fig. 3:**
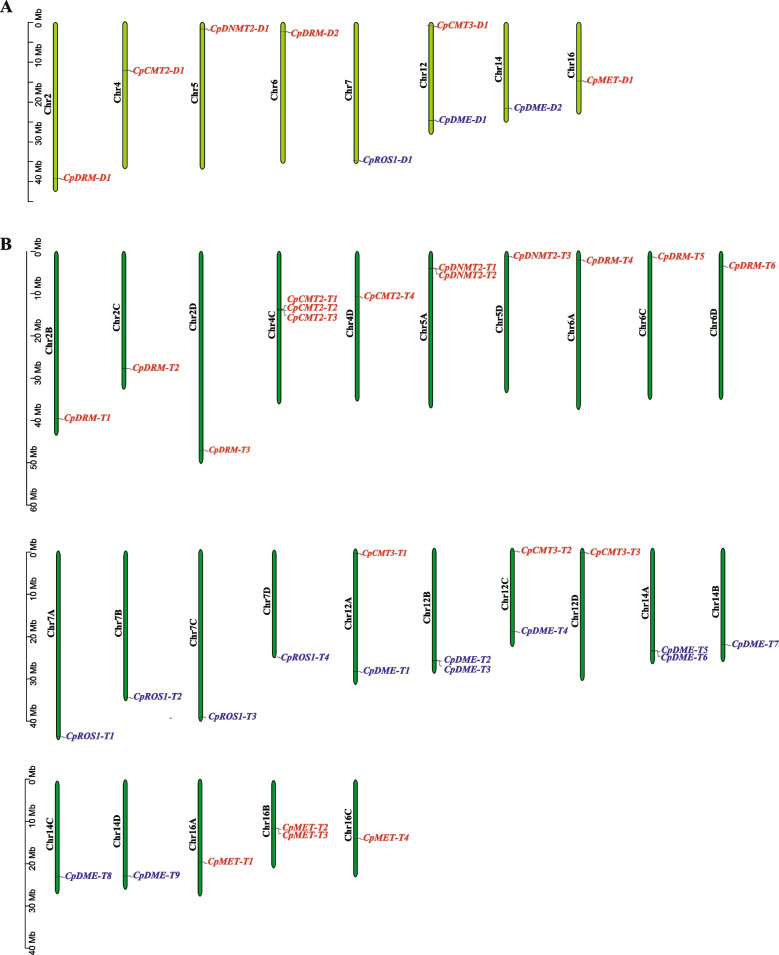
Distribution of *CpC5-MTase* and *CpdMTase* genes on chromosomes in diploid (**A**) and autotetraploid (**B**) *C. paliurus*. Red for *CpC5-MTase* and blue for *CpdMTase*

For diploid *C. paliurus* (2n = 2 ×  = 32), 6 *CpC5-MTase* genes were located on 6 chromosomes (Chr2, Chr4, Chr5, Chr6, Chr12, and Chr16) and 3 *CpdMTase* genes on 3 chromosomes (Chr7, Chr12, and Chr14) (Fig. 3A). For autotetraploid (2n = 4 ×  = 64), 33 identified genes were distributed unevenly in 25 chromosomes, among them, 20 *CpC5-MTase* genes were located on Chr2B/C/D, Chr4C/D, Chr5A/D, Chr6A/C/D, Chr12A/C/D, and Chr16A/C/D; 13 *CpdMTase* genes were located on Chr7A/B/C/D, Chr12A/B/C, and Chr14A/B/C/D (Fig. 3B). Comparatively, Chr4C contained more genes (*CpCMT2-T1/2/3*) than others (only 1 or 2 genes). Noticeably, CpC5-MTase and CpdMTase families were not found in the following 8 pairs of homologous chromosomes 1, 3, 8, 9, 10, 11, 13, and 15. Most genes of *CpC5-MTase* and *CpdMTase* were found to situate near two ends of chromosomes with relatively stable positions both in diploid and autotetraploid *C. paliurus*.

**Fig. 4:**
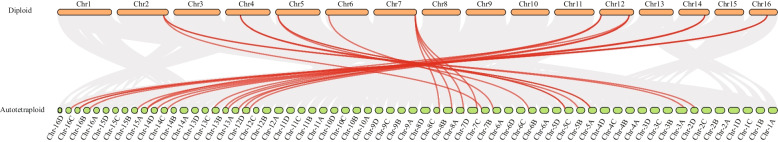
Synteny analysis of *C5-MTase* and *dMTase* genes between two ploidy *C. paliurus.* Gray lines in the background indicate the colinear blocks between diploid (orange) and autotetraploid (green) *C. paliurus*, while the red lines highlight the syntenic *C5-MTase* and *dMTase* gene pairs

Synteny analysis revealed the locus relationship of homologous genes between diploid and autotetraploid *C. paliurus.* As shown in Fig. 4, gene pairs with a collinear relationship were connected with gray lines, while *CpC5-MTase* and *CpdMTase* syntenic gene pairs were linked with red lines. Many chromosomes in both genomes were joined together by lines, indicating that they were evolutionarily related. Also, most genes were conserved during polyploidization, such as *CpC5-MTase* and *CpdMTase*. The duplication types of *CpC5-MTase* and *CpdMTase* genes were mostly WGD or segmental in autotetraploid (Table S1), resulting in the expansion of two families. Furthermore, Ka/Ks ratios were used to estimate the selection pressure among duplicated gene pairs in the autotetraploid genome. The Ka/Ks values of all duplicated *CpC5-MTase* and *CpdMTase* genes pairs were lower than 1.0 (Fig. S2), except for two pairs (*CpCMT2-T2* & *CpCMT2-T4*, *CpDRM-T4* & *CpDRM-T6*), suggesting that most of them may undergo purifying selection [28].

### Gene structure and conserved motif distribution analysis

**Fig. 5:**
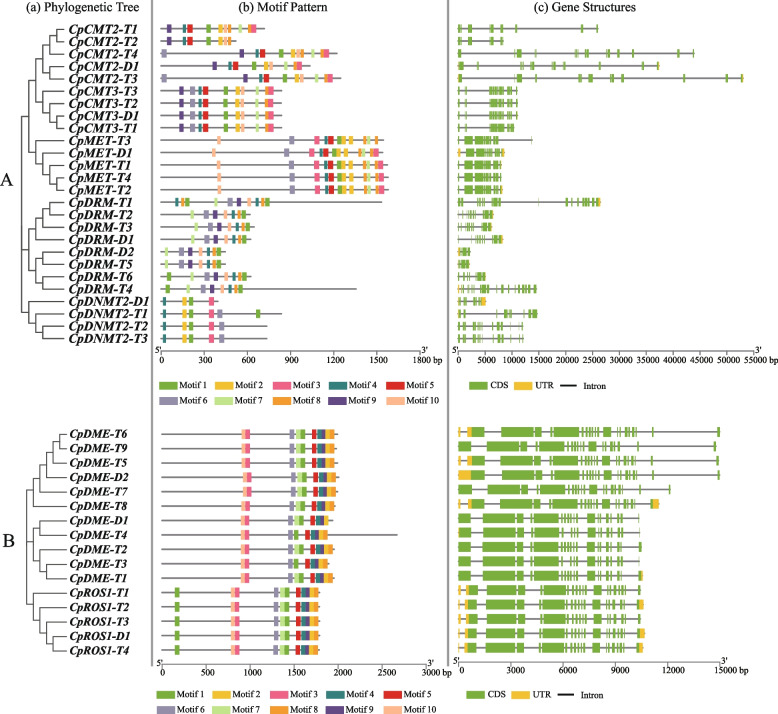
Phylogenetic relationships, conserved motifs, and gene structures of C5-MTase (**A**) and dMTase (**B**) gene families in different ploidy *C. paliurus*. (a) The phylogenetic tree is constructed based on the full-length sequences of C5-MTase or dMTase proteins in different ploidy *C. paliurus* using MEGA 11 software. (b) Motifs pattern of C5-MTase or dMTase proteins. Ten different colored boxes are used to represent the position of different motifs. (c) Gene structure is illustrated with exons represented as green boxes, introns as thin black lines, and UTRs as yellow boxes

As shown in Fig. 5A, different exon–intron distribution patterns were observed in the structure of *CpC5-MTase* in diploid *C. paliurus*. Among them, *CpCMT-Dn* contained the largest number of introns, while *CpCMT2-D1* and *CpCMT3-D1* had 21 and 20 introns, respectively. In other subfamily genes, the number of introns varied from 3 to 11. However, in autotetraploid *C. paliurus,* the ranges of introns in *CpCMT-Tn, CpDRM-Tn*, *CpMET-Tn,* and *CpDNMT2-Tn* were 11–23, 3–20, 11, and 12–16, respectively. Comparatively, the length of intron in *CpC5-MTases* exhibited expansion in autotetraploid, such as *CpCMT2-T3*/4, and *CpDRM-T1*. Furthermore, a significant increment in the number of introns *CpDNMT2-Tn* from autotetraploid was found in comparison with that from diploid. Interestingly, all members of the MET subfamily from both ploidies contain 11 introns, demonstrating their stability during polyploidization. For *CpdMTase* genes (Fig. 5B), the number of introns ranged from 13 to 19 regardless of ploidy. Among them, the least number of introns (13) occurred in *CpDME-T2*. Moreover, *CpROS1s* contained 17–19 introns, indicating their relative conservation in gene structure during polyploidization.

A total of 10 motifs were identified in the CpC5-MTase proteins (Fig. 5A; Table S2). Motifs 1, 2, 4, 5, and 8 located at the C-terminus were highly conserved in CpCMTs and CpMETs*.* All CpDRMs consisted of motifs 1, 4, 6, 7, 8, 9, and 10. Compared to the other three subfamilies, the DNMT2 subfamily had a unique structure, with only motifs 1, 2, 3, and 4 located at the N-terminus. These results may explain the division of CpC5-MTase proteins into three branches, CpCMTs and CpMETs proteins were clustered into a small one, while CpDRMs and CpDNMT2s belonged to separate branches. Similarly, 10 motifs were identified in the CpdMTase proteins, which were grouped into 3 branches (Fig. 5, Table S2). Among these, the structure of the ROS1 subfamily was relatively stable, with the consistent distribution of 10 motifs; all CpDMEs contained all 10 motifs, except for CpDMTE-T3 and CpDMTE-T4 (missing motif 7), CpDME-D1 (missing motif 8).

### Cis-acting element analysis of *CpC5-MTase* and *CpdMTase*

**Fig. 6:**
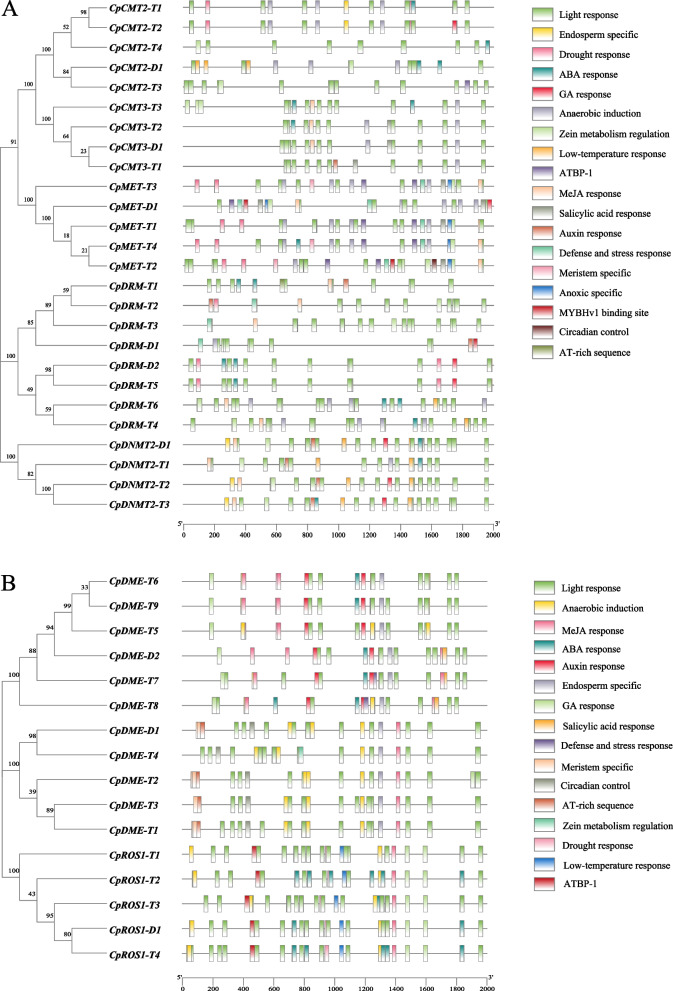
Cis-acting element analysis of *CpC5-MTase* (**A**) and *CpdMTase* (**B**). The analysis is performed on the 2000-bp upstream regulatory regions and evolutionary tree is shown on the left. The scale bar at the base indicates the length of promoter sequence. Different colored boxes represent different cis-responsive elements

In *C. paliurus*, the cis-acting elements in promoter regions of *CpC5-MTase* and *CpdMTase* genes were classified into four categories: light response, phytohormone response, stress response, and plant growth and development (Fig. 6). Notably, each gene was enriched with multiple light-responsive elements. Phytohormone response elements, including ABA, auxin, GA, MeJA, and salicylic acid were also widely observed. Moreover, multiple stress-related elements were also predicted in *CpC5-MTase* and *CpdMTase* genes, implying their role in responding to various stresses, such as anaerobic induction, drought, low temperature, and defense response. In addition, some tissue-specific elements related to endosperm-specific, root, and stem meristem specific were identified. In a word, most of the cis-acting elements found in *CpC5-MTase* and *CpdMTase* were associated with physiological processes, indicating their involvement in plant growth and development, as well as the stress response.

### Expression patterns of *CpC5-MTase* and *CpdMTase* genes in various tissues of diploid *C. paliurus*

**Fig. 7:**
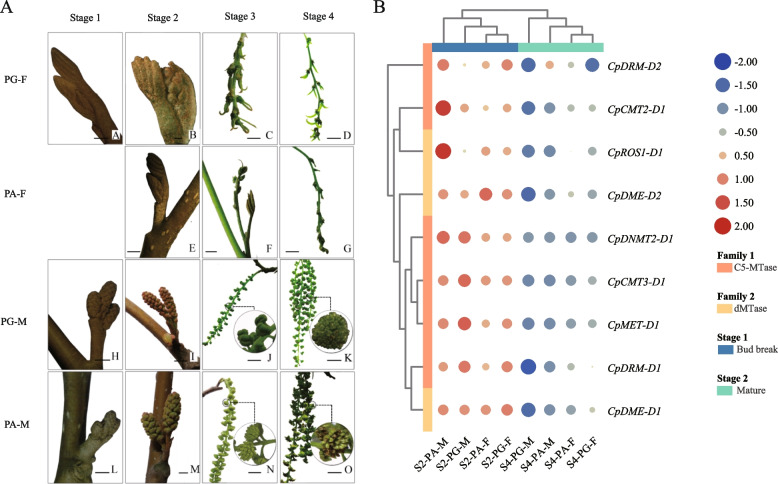
Spatial and temporal expression patterns of *CpC5-MTase* and *CpdMTase* genes in *C. paliurus. ***A** Morphological changes during flower development in *C. paliurus*. S1, dormant stage; S2, bud break stage; S3, inflorescence elongation stage; S4, mature stage. **B** Heat map of *CpC5-MTase* and *CpdMTase* genes expression abundance among different tissues. Blue and red in the color scale indicate low and high transcript expression, respectively. The circle size represents gene expression amounts. PA-F, female floral buds from a protandrous; PA-M, male floral buds from protandrous; PG-F, female floral buds from protogynous; PG-M, male floral buds from protogynous

The flower buds of *C. paliurus* undergo physiological differentiation (S0), dormancy (S1), bud break (S2), inflorescence elongation (S3), and flower maturation stages (S4) (Fig. 7A, cited from [29]). The gene expression patterns of floral buds at the S2 and S4 were analyzed, including female and male buds in protandry (PA) and protogyny (PG) individuals, respectively [30]. The results showed that all *CpC5-MTase* and *CpdMTase* genes were expressed in floral buds. According to heatmap-clustering analysis (Fig. 7B), the expression of all genes was significantly up-regulated at S2 and significantly down-regulated at S4. At S2, the expression levels of *CpCMT3-D1*, *CpDNMT2-D1*, *CpMET-D1*, and *CpDRM-D1* in male floral buds were higher than those in female ones regardless of mating type, indicating that *CpC5-MTases* plays an important role in the morphological development of male flowers. The expression levels of *CpDNMT2-D1*, *CpCMT3-D1*, and *CpMET-1* were higher in PG-M than those in PG-F. In addition, some *CpdMTases* (*CpROS1-D1* and *CpDME-D1*) showed higher expression levels in PA-M than those in PA-F. However, some genes (e.g. *CpDRM-D1*, *CpCMT3-D1* and *CpMET-D1*) displayed the opposite expression pattern in the same sexual floral bud, showing higher expression levels in PG-M versus lower levels in PA-M; whereas *CpDRM-D1* and *CpDME-D1* showed higher expression levels in PG-F than in PA-F. At S4, *CpC5-MTases* showed lower expression levels in male flowers than in female ones regardless of mating type, except that *CpDNMT2-D1* and *CpDRM-D2* expressed higher levels in PA-M than in PA-F. Similarly, the expression levels of *CpdMTase* in male flowers were lower than those in female ones. Moreover, the expression levels of these genes in the same sexual floral bud were also different, such as *CpCMT2-D1*, *CpDRM-D1*, and *CpDME-D1/2* showed higher expression levels in PA-M than in PG-M, *CpROS1-D1* and *CpDME-D2* were expressed higher in PA-F than in PG-F. Thus, we speculate that these genes may fulfill important functions in heterodichogamous characteristics of *C. paliurus*.

### Expression correlation analysis between candidate genes and flowering-related genes

**Fig. 8:**
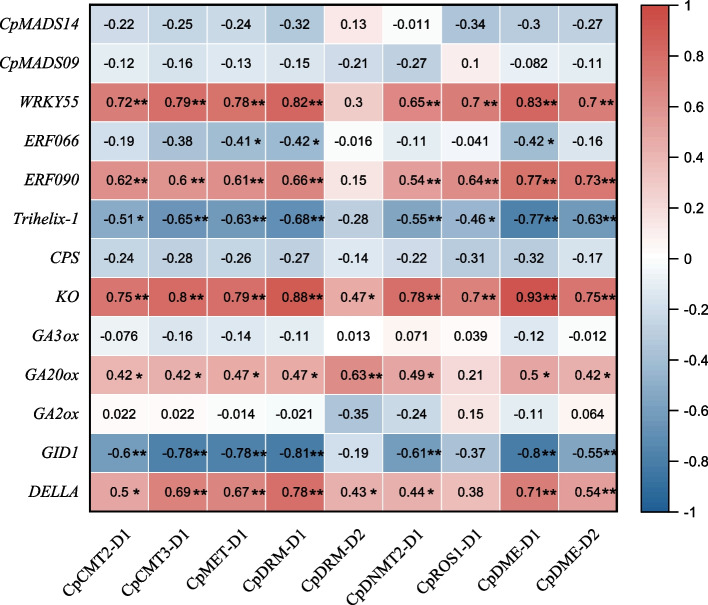
Expression correlation analysis among the candidate genes (*CpC5-MTases* and *CpdMTases*) and flowering-related genes in flowers. Red and blue in the color scale indicate positive correlation and negative correlation, respectively. * and ** represent *P* < 0.05 and *P* < 0.01, respectively

To investigate the potential role of *CpC5-MTases* and *CpdMTases* in heterodichogamy, based on previous research results [17, 23, 30], correlation analysis was performed on the differential genes in the lowering process of PA and PG. Figure 8 illustrated some genes that may play roles in heterodichogamy, such as *ERFs*, *Trihelix-1*, and GA synthesis genes. *CpC5-MTases* and *CpdMTases* were significantly correlated with several genes involved in GA biosyntheses (Fig. 8), including *KO*, *GA20ox*, *GID1*, and *DELLA*. Notably, *CpC5-MTases* showed significant positive correlations with *GA20ox*. Both *CpC5-MTases* and *CpdMTases* were significantly positively correlated with *KO*. With the exception of *CpDRM-D2* and *CpROS-D1*, most of the *CpC5MTases* and *CpdMTases* showed significant negative correlations with *GID1*, but remarkable positive correlation with *DELLA*. Additionally, except for *CpDRM-D2*, most of the *CpC5MTases* and *CpdMTases* were significantly positively correlated with *WRKY55* and *ERF090*, but negatively correlated with *Trihelix-1*.

## Discussion

### Structure and evolutionary features of C5-MTase and dMTase gene families during polyploidization

Homozygous comparisons revealed no differences in the C5-MTase and dMTase gene families between PA-dip and PG-dip genomes (Table S4, Fig. S1-B). Therefore, we further analyzed the genomic data from PA-dip and PA-tetra, identifying 6 *CpC5-MTase* and 3 *CpdMTase* genes in the diploid *C. paliurus,* and 20 *CpC5-MTase* and 13 *CpdMTase* in the autotetraploidy. Compared with reported plants, the members of C5-MTase and dMTase gene families in diploid *C. paliurus* were lower than those in Arabidopsis (11 and 4), rice (7 and 6), and tea plant (8 and 4) [12, 26, 31]. The larger genome in diploid *C. paliurus* (587 Mb) than in rice (466 Mb) and *A. thaliana* (125 Mb) [12, 17], this suggests that gene absence events may have occurred in *CpC5-MTase* and *CpdMTase* genes during the evolution of *C. paliurus*. The phylogenetic tree showed that CMT1 and DML subfamilies were absent in *C. paliurus* compared with other species (Fig. 2). Previous research showed that 73 out of 77 species have experienced gene absence events, such as *CMT1* in sugarcane and *DML* in peanut [32–34]. In Arabidopsis, *AtCMT1* has been proven unessential, as the allotetraploid *A. suecica* contained an Evelknievel retroelement insertion or inactivation of the truncated proteins [35]. Presumably, *CMT1* and *DML* in *C. paliurus* were lost during the evolutionary process due to functional redundancy. However, the effects of gene absence on epigenetic modifications need to be further studied.

Additionally, gene absence commonly occurs during genome sequence rearrangement after chromosome doubling. Qu et al. [17] found a large deletion in Chr8 compared with other chromosomes, and an inversion between Chr4 and Chr16 in the synteny analysis of *C. paliurus* genomes. Similarly, we observed that some genes’ locations changed in chromosomes (Fig. 4), indicating that chromosome rearrangement may exist in the target gene family. Theoretically, based on the ploidy level, the number of *CpC5-MTase* and *CpdMTase* genes in autotetraploid *C. paliurus* should be 36 (9 × 4). Actually, *CpC5-MTase* experienced gene loss during polyploidization. For example, there are 6 *DRM* genes in autotetraploid *C. paliurus*, compared with 8 *DRM* genes in diploidy, suggesting that the DRM subfamily may have undergone copy loss during the evolution of autotetraploid *C. paliurus*. Nonetheless, the number of CpC5-MTase and CpdMTase gene families in autotetraploid (13) was significantly higher than that in diploid *C. paliurus* (3), allotetraploid Brassica napus (6) and peanut (10) [12, 34]. The quantitative advantage demonstrated that the DME subfamily may experience significant expansion during three WGD events, and the autotetraploid speciated in the most recent WGD (~ 11.2–10.5 Mya) [17]. In this study, we found that *CpC5-MTase* and *CpdMTase* genes were mostly involved in WGD and segmental replication (Table S1), which is consistent with the expansion of C5-MTase gene family in autopolyploid sugarcane (*Saccharum spontaneum*) and allopolyploid wheat (*Triticum aestivum*) [33, 36]. Segmented replication often occurs in plants, which experienced polyploidy events resulting in a large number of chromosome blocks in the genome. Therefore, the results confirmed our conjectures that these two gene families experienced gene loss and expansion events during polyploidization, while WGD and segmental replication bring about the increment number of *CpdMTase* genes in autotetraploid *C. paliurus*.

Although the numbers of *C5-MTase* and *dMTase* gene members varied greatly across species, their protein sequences were highly similar. In *C. paliurus*, the C-terminus of CpC5-MTase proteins contained a conserved structural domain of DNA-methylase, but they were equipped with different N-terminal structural domains (Fig. 1), which is similar to the homologous genes in Arabidopsis [12]. The BAH structural domain was presented in all CpMETs and CpCMTs, and it interacted with chromatin to be related to gene silencing and replication. The UBA structural domain, usually as a protein–protein interaction structural domain, was presented in members of the DRM subfamily [37]. In Arabidopsis, normal RNA-directed DNA methylation required both the UBA and DNA-methylase domain [38]. However, both CpDRM-D2 and CpDRM-T5 owned an additional incomplete DNA-methylase domain instead of the UBA domain, which was also reported in peanuts [34]. It is speculated that DRM without a UBA domain may direct DNA de novo methylation upon forming heterodimer with another DRM with a UBA domain [34]. However, the specific function of *CpDRM-D2* and *CpDRM-T5* genes in *C. paliurus* needs to be further explored.

All CpdMTase proteins contained the same conserved structural domains: RRM-DME, Perm-CXXC, and ENDO3c (Fig. 1), which is similar to the homologous genes in Arabidopsis and rapeseed [12]. The ENDO3c domain is the core domain of dMTase, and contains a conserved DNA glycosidase motif that directly removes 5-mC bases [2]. Genes with the same domain, such as *CpDMEs* and *CpROS1*, may exert similar functions in the process of demethylation in *C. paliurus*. Additionally, synteny analysis showed that 80% of analyzed genes situated at the relatively stable positions on the chromosomes (Fig. 4), indicating that the *CpC5-MTase* and *CpdMTase* genes are highly conserved at the DNA level during polyploidization in *C. paliurus*.

Collectively, in both diploid and autotetraploid *C. paliurus*, the *CpC5-MTase* and *CpdMTase* genes were highly conserved not only at the protein level but also at the DNA level, including gene structure, conserved structural domains, as well as the type of motif.

### C5-MTase and dMTase gene families play important roles in regulating the heterodichogamous characteristics in *C. paliurus*

*C. paliurus* is a typical heterodichogamous species, in which the flowering of female flowers in PG synchronizing with male flowers in PA effectively avoids self-pollination [16, 29, 30]. As reported in many plants, *C5-MTase* and *dMTase* genes play important roles in flower development, sex differentiation, and regulation of flowering time [4, 5, 18]. It was documented that the reduction of DNA methylation promoted flowering in Arabidopsis and trifoliate orange (*Poncirus trifoliata*) [39, 40]. However, this is the first report that *C5-MTase* and *dMTase* genes involve in heterodichogamous species. In this study, the expression patterns of 9 candidate genes in counterpart sexual flowers varied across developmental stages (Fig. 7B), presenting their transcript abundances of male and female flowers from two morphs were all higher at S2 than at S4. During the process of floral development in *C. paliurus*, S2 is defined as the pivotal stage affecting further asynchrony [29]. As presented in Fig. 7B, *CpC5-MTases* at S2, including *CpDNMT2-D1*, *CpCMT3-D1,* and *CpMET-D1*, displayed a higher expression level in PG-M, while less or no expression level in PG-F. This suggests that the decrease of DNA methylation in PG-F promotes the development of female prior to male buds at S2, which is in accord with the morphological characters of *C. paliurus* (Fig. 7A). In contrast, *CpdMTases* (*CpROS1-D1* and *CpDME-D1*) were expressed higher in early flowering PA-M than in PA-F at S2 (Fig. 7B), implying that active demethylation in PA-M could promote the development of male prior to female buds at S2, which is in accord with DNA demethylation induces flowering in plants [40]. DNA methylation expresses dynamic changes during flower development [5], and the transcript levels of *CpC5-MTases* and *CpdMTases* decreased with the maturation of male and female flowers in *C. paliuru*s (Fig. 7B). At S4, except *CpDNMT2-D1* and *CpDRM-D2,* the expression levels of *CpC5-MTases* and *CpdMTases* were higher in female than in male flowers regardless of the mating type (Fig. 7B). Moreover, the expression levels of these genes in the same sexual floral bud were also different. Divergent gene expression levels in PA-F/M and PG-F/M at S4 implied that DNA methylation exerts different functions in the morphological development and maturation of male and female flowers. Song et al. [41] identified higher levels of methylation in male than in female flowers in *Populus tomentosa*. Xing et al. [42] argued that the DNA methylation pattern affected gene expression levels in apple buds with different flowering capabilities, to influence the flowering phenotype. Based on the analyzed data from *C. paliurus*, we speculate that the mechanisms of DNA methylation reduction in early flowering between two morphs are probably induced by: a) *DNMT2*, *CMT3,* and *MET* (responsible for establishing and maintaining DNA methylation) being down-regulated in PG-F; b) DNA demethylase *ROS1* being up-regulated in PA-M.

In *C. paliurus*, Qu et al. [23] found that GA_3_ positively regulated the physiological differentiation and germination of floral buds (S2), and GA-related DEGs play central roles in regulating flower development. In addition, Meijón et al. [43] detected that global DNA methylation in azalea treated with a GA biosynthesis inhibitor during the floral transition could promote flowering. While we found that *CpC5-MTases* and *CpdMTases* were significantly associated with several genes related to GA biosynthesis (Fig. 8). It was demonstrated that GA20ox, KO, GID1, and DELLA are the key enzymes in the biosynthesis pathway of gibberellin, and their expression patterns can affect the content of gibberellins [21, 44]. As shown in Fig. 8, *DELLA* and *GID1* were significantly correlated with most *CpC5-MTases and CpdMTases*. DELLA proteins are negative regulators of GA signaling, and DELLA binding to GA receptor GID1 could lead to the degradation of DELLA proteins and the activation of GA function [45, 46]. These key genes in GA biosynthesis and signaling pathway are regulated by epigenetic mechanisms. It has been demonstrated that DELLA protein regulates GA, brassinosteroid and jasmonic acid pathways by histone deacetylation to adjust plant growth and development [47, 48]. *DELLA* displayed a higher expression level in PA-F than that in PA-M at S2, indicating that the DELLA proteins suppress nutritional and reproductive growth [23, 45]. Meanwhile, we identified GA-response CREs in the promoters of these two gene families. Our results suggested that GA may affect the level of DNA methylation by regulating the promoter activities of *CpC5-MTase* and *CpdMTase* genes. Further, Zhang et al. [40] found that GA_3_ participated in the regulation of *CiLFY* (a central regulator of flowering), while demethylation induced *CiLFY* gene expression in trifoliate orange [21]. Accordingly, locus-specific methylation analysis of *CiLFY* showed that the level of DNA methylation decreased during the phase change between juvenile and adult stages in precocious trifoliate orange [40]. Thus, we suggest that the interaction between DNA methylation modifications and gibberellins levels could regulate the asynchronous flowering in heterodichogamous *C. paliurus*. However, the specific regulatory mechanisms need to be further verified. We will further identify the functions of *CpC5MTases* and *CpdMTases* by transient transformation, and the role of DNA methylation modification on the asynchronous floral development process in two morphs will be further revealed by using genome-editing tools.

## Conclusions

We have identified members of C5-MTase and dMTase gene families in diploid and autotetraploid *Cyclocarya paliurus*. WGD or segmental duplication was the main impetus for the expansion of CpdMTase gene families during polyploidization. Furthermore, gene loss events occurred during the speciation of autotetraploid *C. paliurus*, and the *DML* and *CMT1* were missed in comparison with other species. The CpC5-MTase and CpdMTase gene families were highly conserved at the DNA and protein levels during polyploidization. Combined with transcriptome data, we observed differential transcript abundance of *CpC5-MTase* and *CpdMTase* genes in female/male flowers of two morphs (PA and PG) at different stages. Collectively, we speculated that the early flowering of PG-F and PA-M may be caused by the down-regulating expression of CpC5-MTases and the up-regulating expression of CpdMTases, respectively. In addition, *CpC5-MTase* and *CpdMTase* genes were significantly associated with GA synthesis-related genes during flowering. Our results provide novel insights into the molecular mechanisms of heterodichogamy.

## Materials and methods

### Plant materials and transcriptome sequencing

The plants in this study were the diploid *C. paliurus* growing in the Baima experimental base of Nanjing Forestry University, Nanjing, Jiangsu Province, China (31°35′N, 119°09′E). According to the characteristics of floral developmental phase of floral buds [27], female and male floral buds in PA and PG individuals were collected at two stages: 1) S2, bud break stage, flower buds began to protrude; 2) S4, mature stage, the feathery stigma of PG-F opened and the mature pollen of PA-M was released; while the stigma of PA-F was not fully formed, and the anther of PG-M was gradual expansion (Fig. 7A). Each sample contained three biological replicates, the collected samples were frozen immediately in liquid nitrogen and stored at -80 °C before use.

The total RNA was extracted using the E.Z.N.A. plant RNA Kit (Omega, Atlanta, Georgia, USA), and converted into cDNA using the PrimeScript™ II 1st Strand cDNA Synthesis Kit (Takara, Dalian, Liaoning, China). All cDNA libraries were loaded onto an Illumina HiSeq™2000 system (2 × 100 bp read length).

### Identification of the cytosine-5 DNA methyltransferase and demethylase genes

The Hidden Markov Model (HMM) of the DNA methylase domain (PF00145) was downloaded from the Pfam database (http://pfam.xfam.org) to identify the C5-MTase proteins in 3 genomes (PA-dip, PG-dip, and PA-tetra) of *C. paliurus*. Similarly, we downloaded the HMMs of HHH-GPD domain (PF00730) and RRM-DME domain (PF15628) from the Pfam database as the probes to search the dMTase proteins. These HMM profiles were used to perform HMM searches against proteins from *C. paliurus* genome via a local HMMER search program (E-value ≤ 1e^−10^) [49]. The whole protein sequence of *C. paliurus* was obtained based on whole-genome sequencing results [17]. To ensure the accuracy of the predicted genes, the sequences of 11 C5-MTase and 4 dMTase proteins in *Arabidopsis thaliana* were downloaded from the TAIR (https://www.arabidopsis.org) (Table S3) to be used as queries against *C. paliurus* genome database using the local BLAST program (E-value ≤ 0.001) [50], and further compared with conserved domains of the predicted genes using NCBI-CDD (http://ncbi.nih.gov/Structure/cdd/cdd.shtml). After the removal of redundant and incomplete sequences, we obtained the putative C5-MTase and dMTase sequences*.* The new-identified genes were named according to both *A. thaliana* homologs gene and their chromosomal location in *C. paliurus*. Genes from the diploid genome were labeled as “-D” and those from autotetraploid as “-T”.

The ExPASy software (http://web.expasy.org/protparam) was used to analyze the grand average of hydrophobicity (GRAVY), molecular weight (MW), and isoelectric point (pI) of CpC5-MTase and CpdMTase proteins. The subcellular localization of *CpC5-MTase* and *CpdMTase* was further predicted using the WoLF PSORT server (https://wolfpsort.hgc.jp/).

### Phylogenetic tree construction

The MEGA 11 [51] was used to investigate the phylogenetic interactions of C5-MTase and dMTase proteins between *C. paliurus* and other three species *(A. thaliana, O. sativa, and P. trichocarpa)* (Table S3). The protein sequences of the other three species downloaded from both UniProt and NCBI Protein databases, were utilized to construct a phylogenetic tree using MEGA 11 software according to the Maximum Likelihood (ML) method with 1000 bootstrap replicates. Visualization of the phylogenetic tree was accomplished by the Interactive Tree of Life (iTOL) online phylogeny tool [52].

### Chromosome localization and synteny analysis

The distribution information of *CpC5-MTase* and *CpdMTase* genes on the chromosomes of *C. paliurus* was analyzed using TBtools software (GitHub, San Francisco, CA, USA) [53], then named according to their chromosome orders. The synteny relationships of *CpC5-MTase* and *CpdMTase* genes between diploidy and autotetraploidy were exhibited using the TBtools and Multiple Collinearity Scan toolkit (MCScanX) [54]. The nonsynonymous (Ka) and synonymous (Ks) of each duplicated gene pair of two gene families were calculated by the TBtools [28, 53].

### Conserved motif and gene structure analysis

Conserved motifs of all CpC5-MTase and CpdMTase proteins were analyzed using Multiple Expectation Maximization for the Motif Elicitation (MEME, http://meme-suite.org/) [55]. Gene structure analysis was conducted with the Gene Structure Display Server tool (GSDS, http://gsds.cbi.-pku.edu.cn/index.php) [56]. Then, the 2000-bp upstream sequence of *C5-MTase* and *dMTase* genes was extracted by TBtools, and the cis-acting regulatory element was predicted on the PlantCARE website (http://bioinformatics.psb.ugent.be/webtools/plantcare/html/) [53, 57].

### Gene expression analysis

To gain insight into the expression patterns of *CpC5-MTase* and *CpdMTase* in *C. paliurus*, we analyzed transcriptomic data obtained by our research group earlier, including male and female flower buds of both mating types in diploid *C. paliurus* at S2 and S4, respectively [30]. RNA-seq reads were obtained from the Genome Sequence Archive (SRA) database under accession numbers CRA002980 and CRA016788. The expression levels of all transcripts were obtained by calculating fragments per kilobase per million (FPKM) with the StringTie software [58].

### Identification and correlation analysis of genes related to heterodichogamy

*MADS14* (CpaM1st25222) and *MADS09* (CpaM1st23860) were highly expressed in PA-M and PG-F, respectively [26]. *Trihelix-1* (CpaM1st06815), *ERF066* (CpaM1st16083), *ERF090* (CpaM1st01501), *WRKY55* (CpaM1st00114), and some genes related to GA synthesis were found that they have functions in the heterodichogamy characteristics, including *CPS* (CpaM1st44543), *KO* (CpaM1st25482), *GA3ox* (CpaM1st52632), *GA2ox* (CpaM1st19769), *GA20ox* (CpaM1st23650), *GID1* (CpaM1st32729), and *DELLA* (CpaM1st34996) [17, 28]. Pearson correlation coefficient between genes related to heterodichogamy and CpC5-MTase and CpdMTase gene families were analyzed and plotted with Origin software.

## Supplementary Information

**Additional file 1: Fig S1.**

**Additional file 2: Fig S2.**

**Additional file 3: Supplementary tables.**

## Data Availability

The datasets supporting the conclusions of this article are included within the article and its additional files. The flower buds transcriptome raw datasets were deposited in the Genome Sequence Archive (SRA) database (https://ngdc.cncb.ac.cn) under the accession number CRA002980 and CRA016788. The three genome sequences of *C. paliurus* (PA-dip, PG-dip, and PA-tetra) were from the Genome Warehouse at the NGDC, BIG, CAS / CNCB (GWH: GWHBKKW00000000, GWHBKKX00000000, and GWHBKKY00000000), and are publicly accessible at https://ngdc.cncb.ac.cn/gwh/.

## Abbreviations

C5-MTase: Cytosine-5 DNA methyltransferase
dMTase: DNA demethylase
MET1: Methyltransferase 1
CMT2/3: Chromomenthylase 2/3
DRM2: Domain rearranged methyltransferase 2
RdDM: RNA-directed DNA methylation
BAH: Bromo-adjacent homology
DNMT2: DNA methyltransferase 2
DME: Demeter
ROS1: Repressor of silence 1
DML2/3: Demeter-like 2/3
PG: Protogyny
PA: Protandry
S2: Stage of floral bud break
S4: Stage of flower maturation
PG-dip: Protogynous of diploid plant
PA-dip: Protandrous of diploid plant
PA-tetra: Protandrous of autotetraploid plant
PA-F: Female floral buds from a protandrous plant
PA-M: Male floral buds from a protandrous plant
PG-M: Male floral buds from a protogynous plant
PG-F: Female floral buds from a protogynous plant
ORF: Open reading frame; pI: Isoelectric point
GRAVY: Grand average of hydrophobicity
HMM: Hidden Markov Model
MEME: Multiple expectation maximization for motif elicitation
Ka: Non-synonymous substitution rate
Ks: Synonymous substitution rate
FPKM: Fragments per kilobase per million
WGD: Whole-genome duplication
