# Supplementary material for: Genome-wide identification and comparative analysis of DNA methyltransferase and demethylase gene families in two ploidy Cyclocarya paliurus and their potential function in heterodichogamy
Source: BMC Genomics. 2023 May 29;24:287. doi: 10.1186/s12864-023-09383-5 (PMC10226219; doi:10.1186/s12864-023-09383-5)
Supplement: Supplementary file 1 — Additional file 1: Fig S1. [file 12864_2023_9383_MOESM1_ESM.pdf]

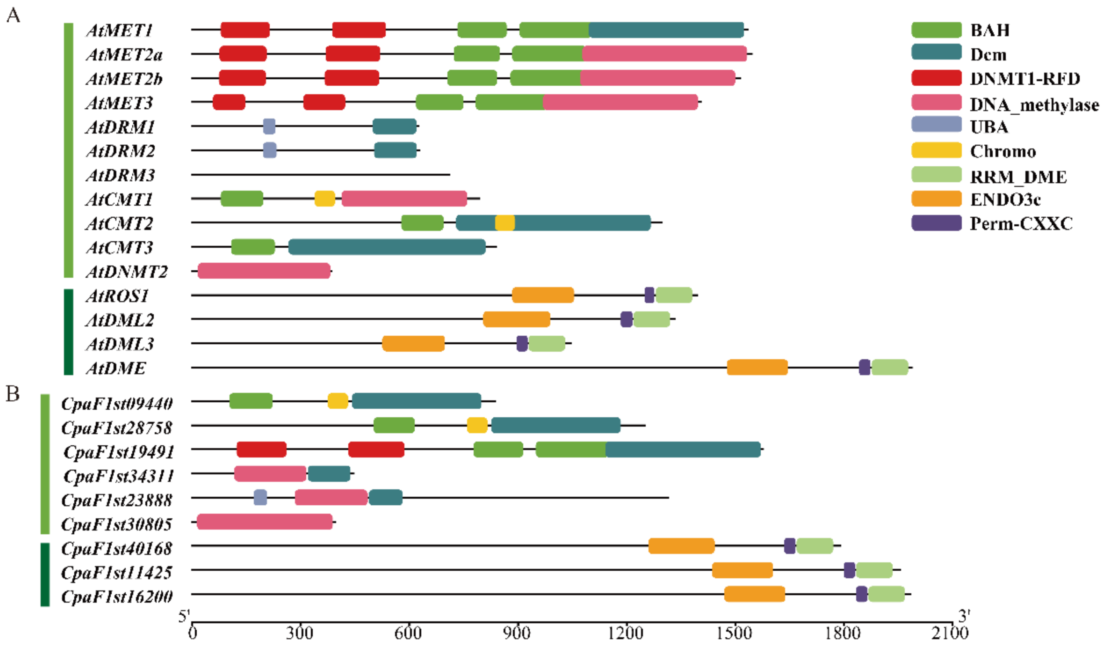

Figure S1. Conserved domain analysis of C5-MTase and dMTase proteins in Arabidopsis (**A**) and dip-PG of *C. paliurus* (**B**).
