# Supplementary figures and images for: Genome-wide identification and comparative analysis of DNA methyltransferase and demethylase gene families in two ploidy Cyclocarya paliurus and their potential function in heterodichogamy
Source: BMC Genomics. 2023 May 29;24:287. doi: 10.1186/s12864-023-09383-5 (PMC10226219; doi:10.1186/s12864-023-09383-5)

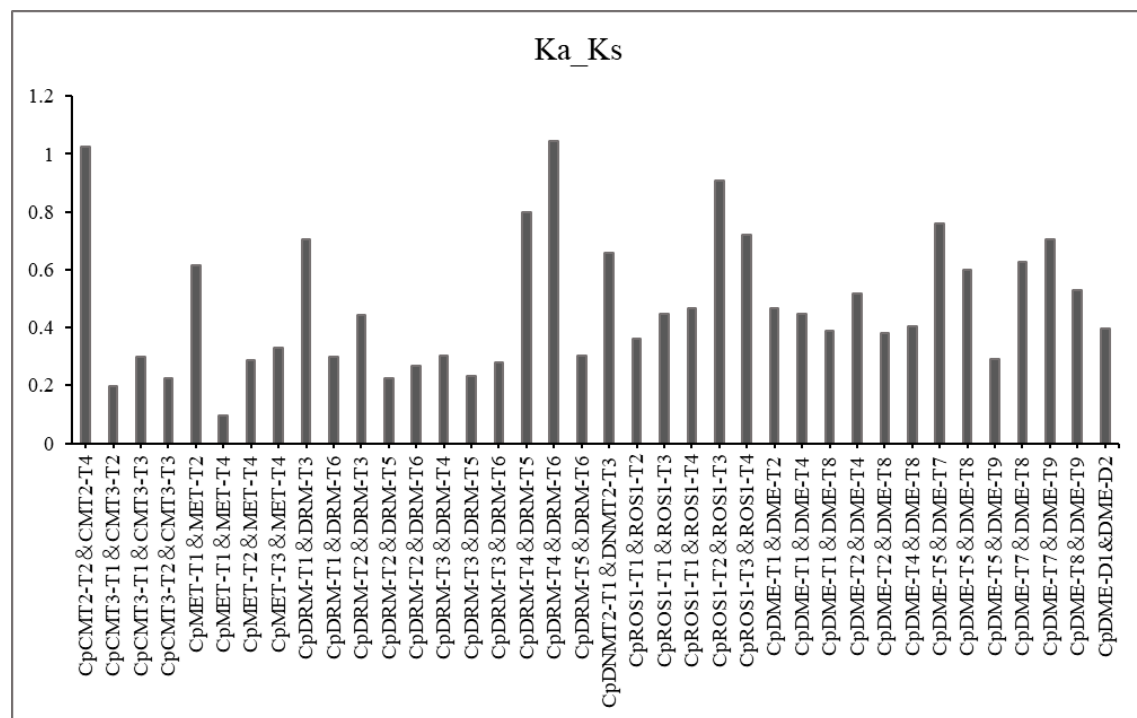

**Figure S2.** Ka/Ks values of duplicated gene pairs of *C5-MTase* and *dMTase* in two ploidy *C. paliurus*.

Supplement: Supplementary file 2 — Additional file 2: Fig S2. [file 12864_2023_9383_MOESM2_ESM.pdf]
